# The Role of Ryanodine Receptor 2 Polymorphisms in Oral Squamous Cell Carcinoma Susceptibility and Clinicopathological Features

**DOI:** 10.3390/ijms251910328

**Published:** 2024-09-25

**Authors:** Ching-Hui Hsu, San-Fu Hong, Yu-Sheng Lo, Hsin-Yu Ho, Chia-Chieh Lin, Yi-Ching Chuang, Ming-Ju Hsieh, Ming-Chih Chou

**Affiliations:** 1 Department of Otorhinolaryngology, Head and Neck Surgery, Changhua Christian Hospital, Changhua 500, Taiwan; 2 Institute of Medicine, Chung Shan Medical University, Taichung 402, Taiwan; 3 Oral Cancer Research Center, Changhua Christian Hospital, Changhua 500, Taiwan; 4 Graduate Institute of Clinical Medicine, College of Medicine, National Chung Hsing University, Taichung 402, Taiwan; 5 Graduate Institute of Biomedical Sciences, China Medical University, Taichung 404, Taiwan; 6 Division of Thoracic Surgery, Department of Surgery, Chung Shan Medical University Hospital, Taichung 402, Taiwan

**Keywords:** head and neck squamous cell carcinomas, oral squamous cell carcinomas, ryanodine receptor 2, single nucleotide polymorphisms

## Abstract

Head and neck squamous cell carcinoma (HNSCC) is the sixth most common malignancy worldwide, and oral squamous cell carcinoma (OSCC) is one of the most common types. There is strong evidence that ryanodine receptor 2 (RYR2) plays an important role in different types of cancer according to previous studies. Its expression is associated with survival in patients with HNSCC, but it is unknown whether altered RYR2 expression contributes to tumorigenesis. Therefore, we examined how RYR2 polymorphisms affect OSCC susceptibility and clinicopathological characteristics. Five single nucleotide polymorphisms (SNPs) of RYR2, rs12594, rs16835904, rs2779359, rs3765097, and rs3820216, were analyzed in 562 cases of OSCC and 332 healthy controls using real-time PCR. We demonstrated that RYR2 SNP rs12594 was significantly different between the case and control groups, but this difference was not significant after adjusting for personal habits. In contrast, we found that different genotypes of SNP rs2779359 were significantly associated with the characteristics of clinical stage and tumor size in OSCC patients, according to the odds ratios and the adjusted odds ratios; specifically, patients with the T genotype had 1.477-fold (95% CI, 1.043 to 2.091; *p* = 0.028) and 1.533-fold (95% CI, 1.087–2.162; *p* = 0.015) increases in clinical stage and tumor size, respectively, compared with patients with the C allele. The results of our study, in which RYR2 SNPs associated with OSCC progression and development were examined for the first time, suggest that clinicopathological characteristics may alter OSCC susceptibility. Finally, RYR2 SNP rs2779359 not only plays a role in both the prognosis and diagnosis of oral cancer but is also likely an important predictive factor for recurrence, response to treatment, and medication toxicity.

## 1. Introduction

Head and neck squamous cell carcinoma (HNSCC) is the sixth most common malignancy worldwide, with oral squamous cell carcinoma (OSCC) being one of its most common types [1]. In certain parts of the world, OSCC represents approximately 90% of all oral malignancies [2,3]. It affects men more frequently than women, and its incidence increases with age. Continued exposure to a number of risk factors, including tobacco, alcohol, betel quid, and human papillomavirus, contributes to the development of oral potentially malignant disease and increases the risk of oral squamous cell carcinoma [4,5]. Even with comprehensive treatment, including surgery, radiation therapy, and chemotherapy, OSCC patients do not have a high survival rate [6]. A dysregulated tumor microenvironment, genetic changes, and epigenetic modifications are all involved in tumorigenesis [7]. Ryanodine receptors (RYRs), which are intracellular calcium (Ca^2+^) channels with high conductivity, control the release of Ca^2+^ from the sarcoplasmic reticulum (a type of endoplasmic reticulum) in certain cells [8]. In RYR2, the amino-terminal, helical, and handle domains drive the interdomains to produce a breathing motion in the overall cytoplasm. Due to the coupling of the U-motif of the central domain with the O-ring of the channel domain formed by the C-terminal domain, the central domain is responsible for the long-range allosteric gating of the channel opening [9,10,11,12]. Previous studies have shown that RYR2 splice variants found in the heart increase susceptibility to apoptosis [13]. Therefore, the RYR2 protein is considered one of the main regulators of cell homeostasis. 

The RYR2 protein, also known as the cardiac ryanodine receptor, is a critical component in the regulation of Ca^2+^ release in cardiac muscle cells [14]. RYR2 is found in the endoplasmic/sarcoplasmic reticulum, a specialized organelle in muscle cells, and plays a crucial role in excitation–contraction coupling (the process by which electrical stimulation triggers muscle contraction) [15,16]. RYR2 regulation is essential for maintaining normal cardiac function, as abnormalities in regulation have been associated with various cardiac disorders, including arrhythmias and cardiomyopathies [17,18]. Cellular Ca^2+^ signaling affects a wide range of processes, and abnormal functioning can contribute to cancer cell proliferation, survival, and metastasis [19]. Alterations in RYR2 expression levels have been implicated in the development and progression of a number of tumor types, including non-small cell lung cancer, colorectal cancer, esophageal cancer, and breast cancer [20,21,22,23]. More specifically, somatic mutations and RYR2 promoter methylation contribute to the pathogenesis of HNSCC by disrupting normal cellular functions and promoting cancer progression [24].

Among the first commonly recognized contributors to human genetic variation were single nucleotide polymorphisms (SNPs), with up to 1% of the approximately 2.9 billion base pairs in the human DNA sequence being potentially different between two individuals [25,26]. SNPs occur in many genes associated with different types of cancer, affecting epigenetic regulation, which increases cancer susceptibility [27]. Polymorphisms in more than 1% of the population result in changes in gene product function that may lead to increased risk of cancer and symptoms of other diseases [28]. SNPs occur in the promoters, exons, and introns of genes and in their 5′ and 3′ untranslated regions (UTRs); they alter gene expression through various mechanisms, thus increasing cancer susceptibility through epigenetic changes [27]. SNPs in exon 37 of the human RYR2 gene result in amino acid exchanges between G1885E and G1886S, which affect the tetrameric channel complex and cause cardiac arrhythmogenesis and failure [29]. The RYR2 SNP rs16835904 T/C genotype promotes the risk of astrocytoma in men, while SNP rs12594 A/G is associated with overall survival in astrocytoma [30]. However, to date, the mechanisms underlying OSCC and the role of RYR2 SNPs have not been elucidated. Therefore, exploring the relationship between RYR2 SNPs and the development of OSCC in Taiwanese individuals could provide valuable information on the genetic mechanisms underlying this cancer.

## 2. Results

### 2.1. Cohort Characteristics

**Figure 1:**
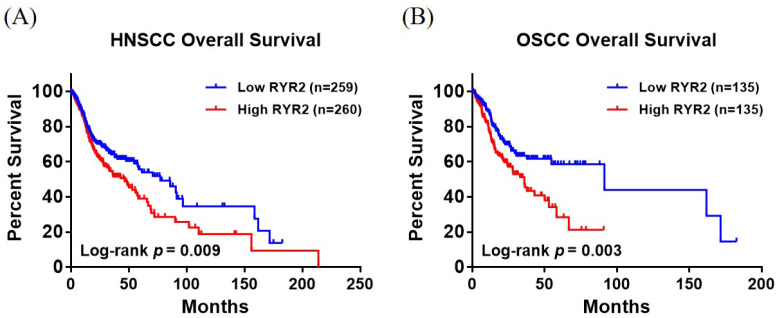
The correlation between the gene expression profile of RYR2 and the clinical outcomes of HNSCC and OSCC patients. (**A**) The overall survival results showed significant differences in HNSCC patients with RYR2 expression. (**B**) OSCC patients with upregulated RYR2 expression had a poor prognosis.

To examine the effect of RYR2 on the progression of HNSCC and OSCC, we used the Xena platform to assess the relationship between RYR2 mRNA at the cell level and patient outcomes from the HNSCC database of The Cancer Genome Atlas (TCGA). The overall survival rate among HNSCC and OSCC patients with high expression levels of RYR2 was significantly lower than that among patients with low levels of RYR2 expression (Figure 1A,B). The results suggest that enhanced expression of RYR2 may play a role in the advancement of both HNSCC and OSCC. In this study, we included 332 healthy controls (noncancerous adults) and 562 patients with OSCC. The two groups did not show significant differences in their mean age based on their demographic, etiological, and clinical characteristics (Table 1). A significant difference in gender distribution was observed between the control group and OSCC patients (*p* < 0.001), which aligns with the current incidence patterns of oral cancer, where it is more prevalent in men than in women. It should be noted that the betel nut-chewing (*p* < 0.001), tobacco-smoking (*p* < 0.001), and alcohol-drinking (*p* < 0.001) groups showed significant differences. The OSCC cohort exhibited all these behaviors more frequently than the controls (Table 1). Approximately two-fifths (39.3%) of OSCC patients had stage I/II cancer and three-fifths (60.7%) had stage III/IV cancer (Table 1). One-third (34.7%) of the cancer cohort had N1-N3 lymph node metastasis. According to our estimates, 96.3% of tumors were identified as M0, and the majority were moderately or poorly differentiated (86.5%) (Table 1).

### 2.2. RYR2 Gene Polymorphism May Affect Mechanisms of OSCC Occurrence and Progression

To investigate the distributions of the RYR2 genotypes, five SNPs were examined, i.e., rs12594 (A/G), rs16835904 (C/T), rs2779359 (C/T), rs3765097 (T/C), and rs3820216 (A/G), in patients with and without OSCC. Based on the logistic regression model, the ORs with their 95% confidence intervals (CIs) were estimated for statistical analysis. As a secondary analytical objective, we combined different variables, including drinking, betel nut chewing, and smoking habits, and the results showed that the OSCC risk in patients with the RYR2 polymorphism rs12594, relative to wild-type individuals, was significantly different (Table 2). According to the rs12594 subgroup, people carrying the GG allele had an increased risk of oral cancer compared with people carrying the AA allele (AA versus GG; OR: 1.956; 95% CI: 1.100-3.481). Additionally, when the model was adjusted for personal habits, there were no significant differences between rs12594 GG and AA allele carriers. We next performed an analysis to determine whether RYR2 SNPs could be associated with OSCC patient characteristics such as clinical stage, tumor size, lymph node metastases, distant metastases, and cell differentiation. We also used multiple logistic regression models to calculate the adjusted odds ratio (AOR) between the two groups after adjusting for betel quid, alcohol, and tobacco consumption. Based on the odds ratio (OR) and AOR estimates (Table 3), we found no significant associations between different genotypes of SNP rs12594 and the various clinicopathological characteristics of patients with OSCC. Consequently, the distribution of different genotypes does not seem to affect OSCC development, progression, or tumor characteristics. In contrast, we found that different genotypes of SNP rs2779359 were significantly associated with the characteristics of clinical stage and tumor size in OSCC patients. Patients carrying the CT + TT allele showed significantly higher incidence of advanced clinical stages (OR: 1.45, 95% CI, 1.32–2.64 [*p* = 0.021]; AOR: 1.477, 95% CI, 1.043–2.091 [*p* = 0.028]) (Table 4) and larger tumor sizes than those carrying the CC allele (OR: 1.517, 95% CI, 1.077–2.137 [*p* = 0.017]; AOR: 1.533, 95% CI, 1.087–2.162 [*p* = 0.015]). However, RYR2 SNP rs2779359 was also related to oral cancer progression in a subgroup of patients with specific clinical stage and tumor size.

### 2.3. Clinical and Functional Insights into OSCC Derived from RYR2

**Figure 2:**
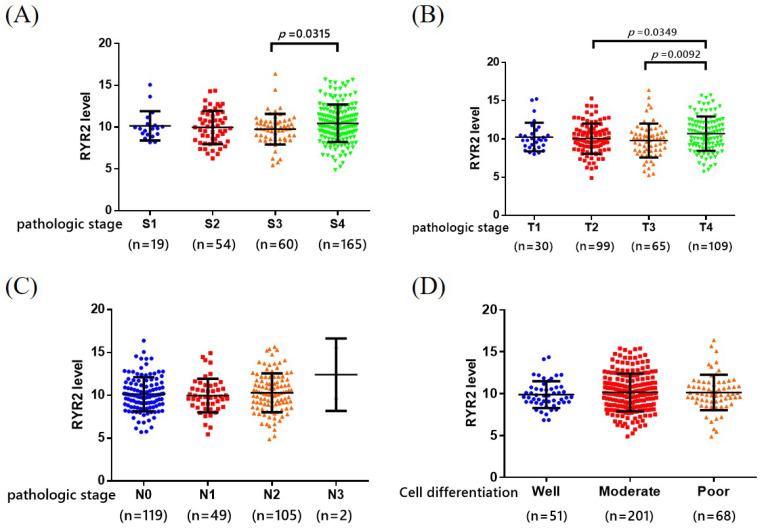
High expression of RYR2 is associated with clinical manifestations in patients with OSCC. The comparisons of RYR2 expression according to (**A**) the pathological stage (blue for stage I, red for stage II, orange for stage III, and green for stage IV), (**B**) the tumor size status (blue for T1, red for T2, orange for T3, and green for T4), (**C**) the lymph node metastasis status (blue for no lymph nodes metastasis, red for N1, orange for N2, and green for N3), and (**D**) the histological grade status (blue for well differentiation, red for moderate differentiation, and orange for poor differentiation). The results with a *p*-value lower than 0.05 were considered statistically significant.

We evaluated the clinical results of the carriers of the RYR2 gene and OSCC patients in the TCGA database. OSCC patients in pathological stage IV showed significantly higher levels of RYR2 than those in other pathological stages (*p* = 0.0315) (Figure 2A). Additionally, higher RYR2 levels were also detected in patients with T4 cancer than those with T2 and T3 cancer (*p* = 0.0349 and 0.0092, respectively) (Figure 2B). However, there were no significant differences between RYR2 levels and lymph node metastasis, nor between RYR2 levels and histologic grade (Figure 2C,D). The findings demonstrate the importance of the pathological stage and tumor size in the genetic association between the expression of the RYR2 gene and OSCC.

## 3. Discussion

We previously noted that RYR2 could be related to the occurrence and prognosis of various cancers. In particular, high expression of RyR2 in colorectal cancer patients has been found to result in shorter survival times [21]. By using data from the TCGA database, we evaluated RYR2’s predictive performance in head and neck cancer, especially oral cancer. Compared with patients with low RYR2 expression, OSCC patients with high RYR2 expression exhibited significantly shorter overall survival (Figure 1B). Based on our findings above, RYR2 may be considered a risk factor for the prognosis of OSCC. Currently, there is no clear correlation between RYR2 polymorphisms and OSCC risk factors, despite extensive research on the relationship between RYR2 alleles and disease susceptibility. Consequently, our findings provide new insights into how RYR2 SNPs contribute to OSCC susceptibility and clinicopathological conditions. 

There is strong evidence from multiple studies that oral cancer is linked to exposure to environmental carcinogens, including smoking, drinking alcohol, and chewing betel nuts [31,32]. As a result of this case–control study, we found that the habits of smoking, eating betel nuts, and drinking alcohol differed significantly between the two groups (Table 1). RYR2 mutations affect Ca^2+^ signaling pathways, which are crucial for cell proliferation and metastasis, the most important factors in the pathogenesis of various cancers [33]. As a non-coding region of mRNA, RYR2 rs12594 is located in the 3′-UTR, but SNPs within this region can be degraded, translated, and localized [34]. Researchers have previously found that the G allele of rs12594 may increase bleeding risk in patients taking direct oral anticoagulants as a result of altered RYR2 expression [35]. In this study, we compared the odds ratios between the controls and OSCC patients and found that they significantly differed in the RYR2 G allele of rs12594 but not after adjusting the ORs for personal habits (Table 2). There may be a relationship between oral cancer risk and the RYR2 rs12594 variant in the context of consumption behaviors. However, further estimation of the OR and AOR revealed that different genotypes of SNP rs12594 were not significantly associated with various clinicopathological characteristics in OSCC patients (Table 3). Previous studies have shown that genetic variation plays an important role in cancer progression and prognosis, as evident from the correlation of SNPs with OSCC tumor size and stage [36,37]. In particular, patients with OSCC who carried the MALAT1 rs619586 polymorphism showed significant differences in both clinical stage and tumor size [38]. There was a significant correlation between RYR2 rs2779359 and the clinical status of oral cancer patients in our study. Compared with patients with the SNP rs2779359 C genotype, patients with the T genotype were in a significantly more advanced clinical stage and presented a significantly larger tumor size according to the ORs and AORs (Table 4).

Further analysis was performed using TCGA database to evaluate the clinical outcomes of RYR2 gene carriers and OSCC patients. In OSCC, RYR2 gene expression correlated with clinical stage and tumor size (Figure 2). As a result of this study, it was also observed that RYR2 SNP rs2779359 is related to the clinicopathological characteristics mentioned above, especially when it comes to advanced clinical stages and large tumor sizes. This SNP may thus be considered a key marker of tumor recurrence, response to targeted therapy, and drug toxicity in patients with oral cancer, according to our findings. Further research is needed to understand the link between RYR2 SNP rs2779359 and other common somatic genetic changes in oral cancer.

There are some limitations to our study that deserve to be mentioned. First, neither the Taiwanese database nor the samples in this study were representative enough to determine the disease pathways for the oral cancer–RYR2 association the wider population. It is essential to confirm our findings on RYR2 SNPs in ethnically diverse OSCC cohorts. Other elements that are equally important for long-term survival are the willingness and recall of the patients. To determine whether the reported RYR2 SNP is related to OSCC, more studies with larger sample sizes and longer follow-ups are needed.

## 4. Materials and Methods

### 4.1. Patients and Specimens

The collection of data on 562 oral cancer cases and 332 healthy controls from 2013 to 2023 was approved by the Institutional Review Board (IRB) of Changhua Christian Hospital (CCH) in Taiwan (study CCH IRB approval No. 130616) and CCH Biobank (IRB No. 200211). The healthy controls (noncancerous adults) were randomly selected from the physical examination center of Changhua Christian Hospital. All study participants (894 cases in total) signed an informed consent form prior to participation in the study. Statistical information on age and personal habits, including the consumption of betel nuts, tobacco, and alcohol, was obtained from medical records. Betel quid and alcohol consumption was defined as constantly chewing betel quid or drinking alcohol, respectively, and smoking was classified as more than one cigarette per day in the previous three months. In this study, we used the standard TNM staging system of the American Joint Committee on Cancer (AJCC) for determining clinical characterization, lymph node metastasis, and tumor cell differentiation in OSCC [39]. For the analysis of RYR2 polymorphisms, the researchers collected venous blood samples and stored them in tubes containing acidic citric dextrose (ACD) solution, which were then cryogenically centrifuged and stored at a temperature of −80 °C in a laboratory freezer.

### 4.2. Bioinformatic Analysis of RYR2 Expression

In this study, we downloaded data from the TCGA database using Xena functional genomics explorer (University of Santa Cruz, California (UCSC)), which allowed us to examine the expression of RYR2 in association with the clinical characteristics of HNSCC and OSCC. Additionally, users of the UCSC Xena browser website (https://xenabrowser.net/) (accessed on 23 January 2024) could access and analyze various genome datasets [40]. 

### 4.3. Functional Selection of RYR2 SNPs

In this study, RYR2 SNPs were selected using the ABI SNP browser, and linkage disequilibrium (LD) emphasized through the LD link website was excluded. We also excluded minor allele frequencies (MAFs) with few genetic sites, i.e., those that exceeded 0.8 and had a minimum of 10% MAF, using the National Institutes of Health Variation Viewer [41,42,43,44]. Based on these studies, five RYR2 SNPs, rs12594 (A/G), rs16835904 (C/T), rs2779359 (C/T), rs3765097 (T/C), and rs3820216 (A/G), associated with many diseases were included in the analysis model [30,45,46,47]. The RYR2 variants rs12594 and rs16835904 are located in the 3’-UTR, while rs2779359 is situated in an intronic region. Additionally, rs3765097 and rs3820216 are classified as synonymous variants. Each of these genotyping experiments was performed by Applied Biosystems using microbial genotyping tests with a mixture of TaqMan minor groove (MGB) probes. The probe identification results of the TaqMan-SNP genetic analysis data sheet included rs12594 (C_8859469), rs168359904 (C_32744084), rs2779359 (C_15932201), rs3765097 (C_26180871), and rs3820216 (C_27490319), which were all stored at −80 °C. In each TaqMan-MGB genotyping mixture, the variant of the wild-type sequence marked VIC corresponded to the variant of the six-carboxyfluorescence (FAM) mutation sequence [48].

### 4.4. DNA Extraction and Analysis of RYR2 SNPs with Real-Time PCR

As in previous studies [49,50], we used DNA extraction, preservation, and analysis techniques to ensure DNA availability. We collected blood samples from all patients into sterile tubes with an ACD solution, which were immediately centrifuged and stored at -80 °C. Genomic DNA was extracted from peripheral blood leukocytes using a QIAamp DNA blood mini kit and then dissolved in TE buffer and stored at −20 °C. Quantification was based on the measurement of the optical density at a wavelength of 260 nm. The five polymorphisms of the potential RYR2 gene, rs12594 (A/G), rs16835904 (C/T), rs2779359 (C/T), rs3765097 (T/C), and rs3820216 (A/G), were determined with quantitative real-time PCR using an ABI StepOne real-time PCR system (Applied Biosystems, Foster City, CA, USA) and were analyzed using the StepOne software (version 2.3). For each reaction, 2.5 µL of TaqMan genotyping master mix was combined with 0.125 µL of TaqMan probe mix and 30 ng of genomic DNA, resulting in a volume of 5 µL. For real-time PCR, the initial denaturation step was conducted at 95 °C for 10 min, followed by 40 amplification cycles at 95 °C for 15 sec and 60 °C for 1 min.

### 4.5. Statistical Analysis

In our study, we performed analyses similar to those in previous papers and used IBM SPSS statistics (version 22.0; IBM, Armonk, NY, USA) [51]. To assess the differences between the OSCC and healthy groups based on demographic and laboratory data, our first step was to use descriptive analyses, including means, standard deviations (SDs), and percentages, and Mann–Whitney U tests. We then used logistic regression models to estimate the odds ratios (ORs) and 95% confidence intervals (CIs) of the RYR2 polymorphism distributions in the healthy and OSCC populations. The AORs between the two groups were calculated after adjusting for betel nut, alcohol, and tobacco consumption and using multiple logistic regression models. The AORs with 95% CI were then generated by analyzing the correlation between RYR2 SNPs (rs12594 and rs2779359) and the clinicopathological characteristics of OSCC. An analysis of the variation in RYR2 levels in the TCGA HNSCC dataset was performed using the Mann–Whitney U test. In order for differences to be considered statistically significant, a *p*-value lower than 0.05 was required.

## 5. Conclusions

In conclusion, our experimental results confirm an association between SNP rs2779359 in the RYR2 gene and OSCC, as well as with advanced pathological stages and large tumor sizes. Patients with the SNP T genotype had 1.477-fold and 1.533-fold increases in clinical stage and tumor size compared with patients with the C allele, respectively, according to the AORs. Therefore, this genetic variation has the potential to serve as a biomarker for OSCC, allowing people at increased risk to be identified earlier and have their progression monitored. Finally, RYR2 SNP rs2779359 not only plays a role in both the prognosis and diagnosis of oral cancer but is also likely an important predictive factor for recurrence, response to treatment, and medication toxicity.

## Figures and Tables

**Table 1:** The distribution of demographic characteristics and clinical parameters of 332 controls and 562 cases with OSCC.

Variable	Controls (N = 332)	Patients (N = 562)	*p*-Value
Age (years)			
>54	169 (50.9%)	248 (44.1%)	0.057
≤54	163 (49.1%)	314 (55.9%)	
Gender			
Male	196 (59.0%)	546 (97.2%)	<0.001 *
Female	136 (41.0%)	16 (2.8%)	
Betel nut chewing			
No	315 (94.9%)	189 (33.6%)	<0.001 *
Yes	17 (5.1%)	373 (66.4%)	
Cigarette smoking			
No	307 (92.5%)	120 (21.4%)	<0.001 *
Yes	25 (7.5%)	442 (78.6%)	
Alcohol drinking			
No	323 (97.3%)	365 (64.9%)	<0.001 *
Yes	9 (2.7%)	197 (35.1%)	
Stage			
I + II		221 (39.3%)	
III + IV		341 (60.7%)	
Tumor T status			
T1 + T2		248 (44.1%)	
T3 + T4		314 (55.9%)	
Lymph node status			
N0		367 (65.3%)	
N1 + N2 + N3		195 (34.7%)	
Metastasis			
M0		541 (96.3%)	
M1		21 (3.7%)	
Cell differentiation			
Well differentiated		76 (13.5%)	
Moderately or poorly differentiated		486 (86.5%)	

N: number. * *p*-Value < 0.05 indicates statistical significance.

**Table 2:** The distribution of genotype frequencies in RYR2 SNPs in control and OSCC groups.

Variable	Controls (N = 332)	Patients (N = 562)	OR ^a^ (95% CI)	*p*-Value	AOR ^b^ (95% CI)	*p*-Value
rs12594						
AA	187 (56.3%)	298 (53.0%)	1.000 (reference)		1.000 (reference)	
AG	128 (38.6%)	211 (37.5%)	1.034 (0.777–1.377)	0.816	1.113 (0.739–1.676)	0.608
GG	17 (5.1%)	53 (9.5%)	1.956 (1.100–3.481)	0.022 *	1.889 (0.858–4.157)	0.114
AG + GG	145 (43.7%)	264 (47.0%)	1.143 (0.870–1.501)	0.339	1.203 (0.814–1.778)	0.353
rs16835904						
CC	159 (47.9%)	277 (49.3%)	1.000 (reference)		1.000 (reference)	
CT	142 (42.8%)	248 (44.1%)	1.002 (0.755–1.332)	0.986	0.834 (0.555–1.252)	0.380
TT	31 (9.3%)	37 (6.6%)	0.685 (0.409–1.147)	0.150	0.542 (0.250–1.176)	0.121
CT + TT	173 (52.1%)	294 (50.7%)	0.946 (0.721–1.241)	0.686	0.781 (0.529–1.154)	0.215
rs2779359						
CC	130 (39.2%)	215 (38.3%)	1.000 (reference)		1.000 (reference)	
CT	157 (47.3%)	262 (46.6%)	1.009 (0.752–1.354)	0.952	0.917 (0.602–1.396)	0.686
TT	45 (13.6%)	85 (15.1%)	1.142 (0.749–1.742)	0.537	0.921 (0.501–1.692)	0.791
CT + TT	202 (60.8%)	346 (61.6%)	1.031 (0.780–1.362)	0.830	0.918 (0.617–1.365)	0.671
rs3765097						
TT	165 (49.7%)	278 (49.5%)	1.000 (reference)		1.000 (reference)	
TC	131 (39.5%)	229 (40.7%)	1.038 (0.778–1.384)	0.802	1.251 (0.828–1.889)	0.287
CC	36 (10.8%)	55 (9.8%)	0.907 (0.571–1.440)	0.678	0.958 (0.487–1.883)	0.901
TC + CC	167 (50.3%)	284 (50.5%)	1.009 (0.769–1.324)	0.946	1.187 (0.804–1.754)	0.389
rs3820216						
AA	284 (85.5%)	466 (82.9%)	1.000 (reference)		1.000 (reference)	
AG	44 (13.3%)	90 (16.0%)	1.247 (0.844–1.840)	0.267	1.410 (0.816–2.437)	0.218
GG	4 (1.2%)	6 (1.1%)	0.914 (0.256–3.267)	0.890	2.101 (0.459–9.626)	0.339
AG + GG	48 (14.5%)	96 (17.1%)	1.219 (0.836–1.776)	0.303	1.466 (0.868–2.476)	0.153

N: number. ^a^ The ORs and their 95% confidence intervals were estimated using logistic regression models. ^b^ The AORs and their 95% confidence intervals were estimated using multiple logistic regression models after controlling for betel nut, alcohol, and tobacco consumption. * *p*-Value < 0.05 indicates statistical significance.

**Table 3:** The clinical status and RYR2 rs12594 genotype frequency in the OSCC group.

Variable	RYR2 (rs12594)					
	AA (%) (N = 298)	AG + GG (%) (N = 264)	OR ^a^ (95% CI)	*p*-Value	AOR ^b^ (95% CI)	*p*-Value
Clinical stage						
Stage I/II	112 (37.6%)	109 (41.3%)	1.000	0.370	1.000	0.383
Stage III/IV	186 (62.4%)	155 (58.7%)	0.856 (0.610–1.202)		0.860 (0.612–1.207)	
Tumor size						
T1 + T2	131 (44.0%)	117 (44.3%)	1.000	0.932	1.000	0.951
T3 + T4	167 (56.0%)	147 (55.7%)	0.986 (0.706–1.376)		0.990 (0.708–1.383)	
Lymph node metastasis						
No	190 (63.8%)	177 (67.0%)	1.000	0.414	1.000	0.425
Yes	108 (36.2%)	87 (33.0%)	0.865 (0.610–1.226)		0.867 (0.610–1.231)	
Distant metastasis						
No	287 (96.3%)	254 (96.2%)	1.000	0.952	1.000	0.971
Yes	11 (3.7%)	10 (3.8%)	1.027 (0.429–2.459)		1.016 (0.424–2.437)	
Cell differentiation						
Good	35 (11.7%)	41 (15.5%)	1.000	0.192	1.000	0.190
Moderate/poor	263 (88.3%)	223 (84.5%)	0.724(0.446–1.176)		0.722 (0.444–1.175)	

N: number. ^a^ The ORs and their 95% confidence intervals were estimated using logistic regression models. ^b^ The AORs and their 95% confidence intervals were estimated using multiple logistic regression models after controlling for betel nut, alcohol, and tobacco consumption.

**Table 4:** The clinical status and RYR2 rs2779359 genotype frequency in the OSCC group.

Variable	RYR2 (rs2779359)					
	CC (%) (N = 216)	CT + TT (%) (N = 346)	OR ^a^ (95% CI)	*p*-Value	AOR ^b^ (95% CI)	*p*-Value
Clinical stage						
Stage I/II	97 (44.9%)	124 (35.8%)	1.000	0.033 *	1.000	0.028 *
Stage III/IV	119 (55.1%)	222 (64.2%)	1.459 (1.032–2.064)		1.477 (1.043–2.091)	
Tumor size						
T1 + T2	109 (50.5%)	139 (40.2%)	1.000	0.017 *	1.000	0.015 *
T3 + T4	107 (49.5%)	207 (59.8%)	1.517 (1.077–2.137)		1.533 (1.087–2.162)	
Lymph node metastasis						
No	142 (65.7%)	225 (65.0%)	1.000	0.863	1.000	0.796
Yes	74 (34.3%)	121 (30.5%)	1.032 (0.722–1.475)		1.049 (0.732–1.502)	
Distant metastasis						
No	209 (96.8%)	332 (96.0%)	1.000	0.625	1.000	0.618
Yes	7 (3.2%)	14 (4.0%)	1.259 (0.500–3.171)		1.265 (0.502–3.192)	
Cell differentiation						
Good	28 (13.0%)	48 (13.9%)	1.000	0.759	1.000	0.810
Moderate/poor	188 (87.0%)	298 (86.1%)	0.925 (0.561–1.525)		0.940 (0.569–1.554)	

N: number. ^a^ The ORs and their 95% confidence intervals were estimated using logistic regression models. ^b^ The AORs and their 95% confidence intervals were estimated using multiple logistic regression models after controlling for betel nut, alcohol, and tobacco consumption. * *p*-Value < 0.05 indicates statistical significance.

## Data Availability

The datasets generated for this study are available upon request to the corresponding authors.
